# Supplementary material for: FAM83A and FAM83B as Prognostic Biomarkers and Potential New Therapeutic Targets in NSCLC
Source: Cancers (Basel). 2019 May 11;11(5):652. doi: 10.3390/cancers11050652 (PMC6562954; doi:10.3390/cancers11050652)
Supplement: Supplementary file 1 [file cancers-11-00652-s001.pdf]

Supplemantary Materials: FAM83A and FAM83B as Prognostic Biomarkers and Potential New Therapeutic Targets in NSCLC

Sarah Richtmann, Dennis Wilkens, Arne Warth, Felix Lasitschka, Hauke Winter, Petros Christopoulos, Felix J. F. Herth, Thomas Muley, Michael Meister and Marc A. Schneider

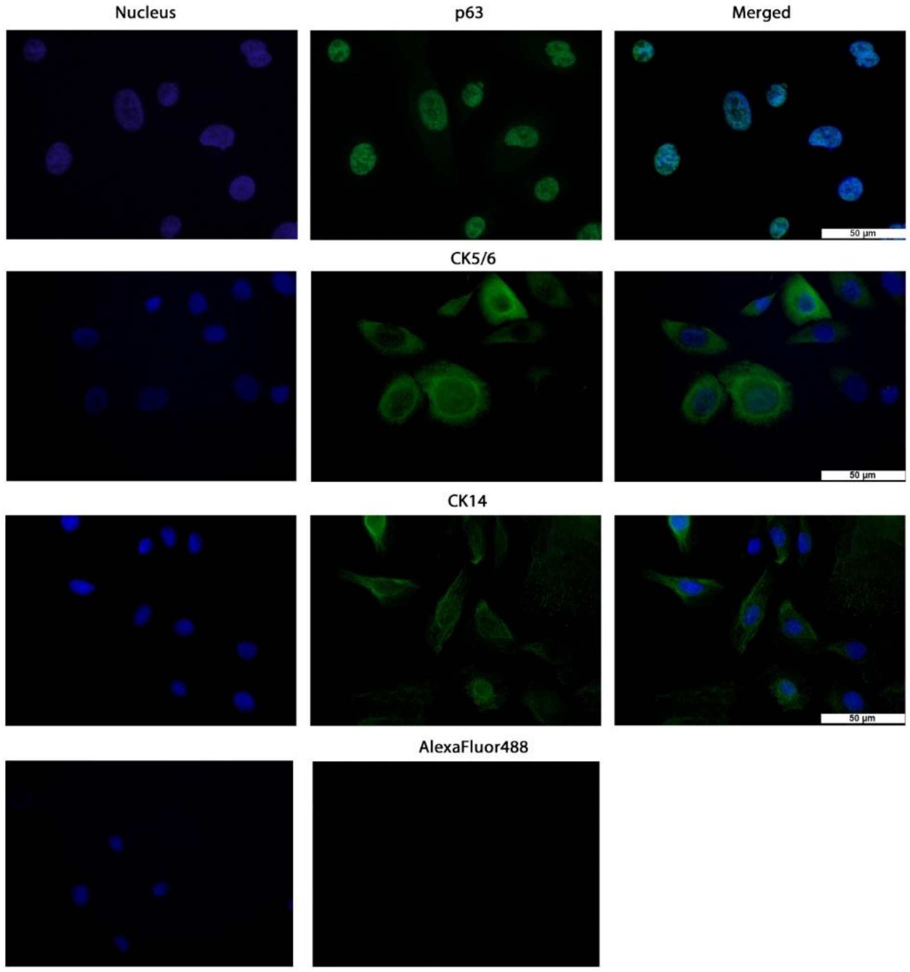

Figure S1. Immunofluorescence in primary cell line 161735T. The markers p63, CK 5/6 and CK14 were tested to validate the primary cell line to be derived from squamous epithelium. As control, only the secondary antibody conjugated to AlexaFluor488 was applied. Scale = 50 µm.

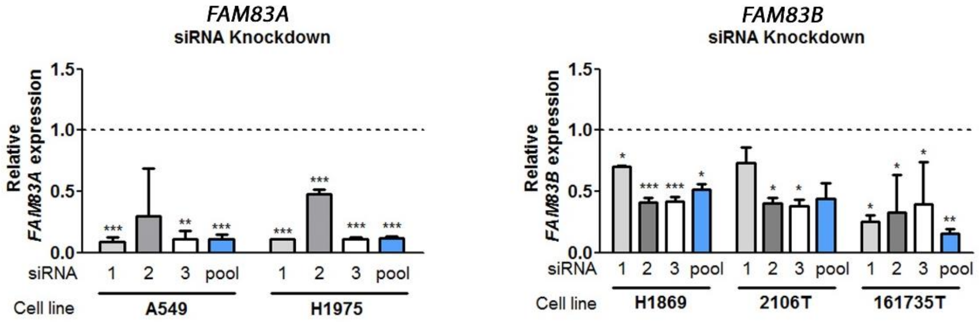

Figure S2. Proof of siRNA mediated knockdown efficiency. Single and pooled siRNAs were tested by qPCR. (*** $p < 0.0001$, ** $p < 0.005$, * $p < 0.05$)

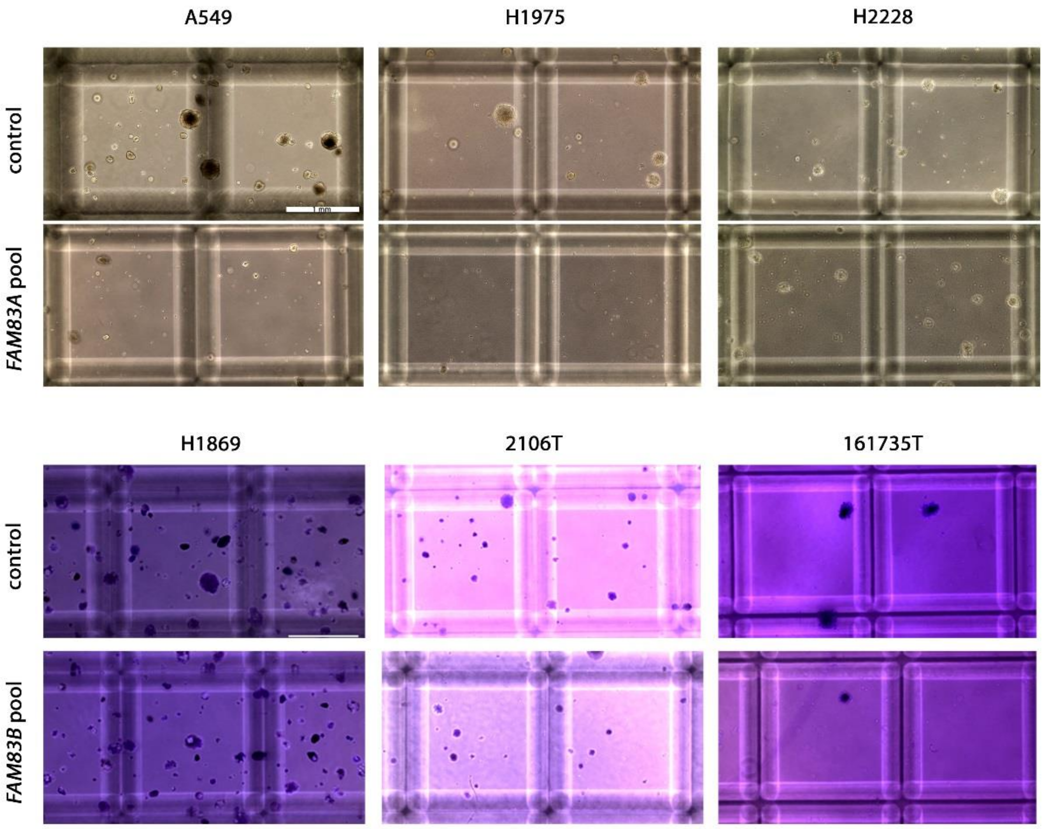

Figure S3. Spheroids for soft agar assay. Spheroids with a diameter of at least 100 μm were counted. Scale = 1 mm.

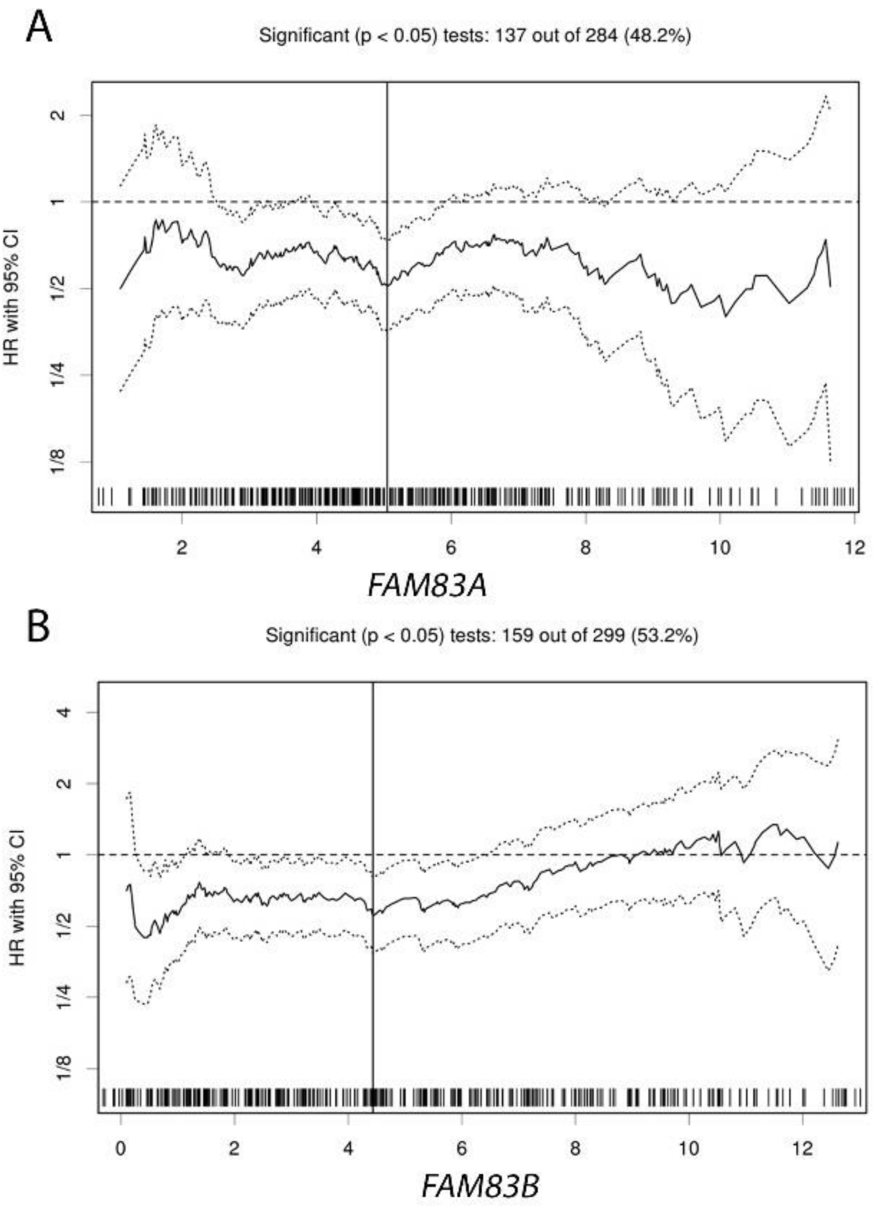

Figure S4. Determination of cut-off for statistical analyses. Images A and B illustrate the respective hazard ratio graphs for *FAM83A* and *B*. Calculated value was used for Cox regression analyses.

© 2019 by the authors. Licensee MDPI, Basel, Switzerland. This article is an open access article distributed under the terms and conditions of the Creative Commons Attribution (CC BY) license (http://creativecommons.org/licenses/by/4.0/).
